# Challenges to the Design of Clinical Trials for Live-Attenuated Tetravalent Dengue Vaccines

**DOI:** 10.1371/journal.pntd.0004854

**Published:** 2016-08-11

**Authors:** Philip K. Russell, Scott B. Halstead

**Affiliations:** 1 Sabin Vaccine Institute, Washington, D.C., United States of America; 2 Department of Preventive Medicine and Biometrics, Uniformed University of the Health Science, Bethesda, Maryland, United States of America

Viral vaccines do not always produce beneficial results. In the 1960s, formalin-inactivated measles and respiratory syncytial viral vaccines established immune responses in recipient children that rendered them susceptible to severe and fatal disease accompanying breakthrough measles or RSV infections [1–3]. It has been understood for some time that dengue vaccines could be subject to a similar outcome. The challenges to the evaluation of any dengue vaccine in clinical trials are uniquely complicated because of the problem of immune enhancement. The vaccine, itself, can raise either a protective or an enhancing immune response or variations in between.

Immune enhancement modifies the response to dengue virus (DENV) infections whether a wild-type virus or a live-attenuated virus. This is due to the observations that a significant contribution to human DENV infections resides in cells of myeloid lineage. A vast majority of antibodies raised to wild-type as well as to attenuated DENV are capable of mediating antibody-dependent enhancement of infection (ADE) in target Fc-receptor–bearing cells [4]. When these antibodies react with a heterologous DENV, the infectious immune complexes formed attach to Fc-receptors, resulting in direct increase in the number of infected cells and the quantity of virus produced per cell [5,6]. While the ADE phenomenon has been documented widely within the Flavivirus group, in vitro, in humans, in vivo, the phenomenon is restricted to DENV. Epidemiologic and clinical studies demonstrated that ADE is a major factor in the pathogenesis of severe DENV disease. Monotypic dengue immunes are at higher risk of dengue hemorrhagic fever and dengue shock syndrome than non-immunes of the same age [7]. Pre-existing Japanese encephalitis antibody is associated with an increase from inapparent to mild overt DENV disease in Thai children [8]. The presence of antibody due to yellow fever vaccination was shown to enhance antibody and viremia responses to an early live-attenuated DENV 2 vaccine [9]. Thus immune enhancement is a well-established mechanism that cannot be ignored in the clinical evaluation of dengue vaccines.

Effective protective immunity to challenge with homologous or heterologous DENV results from components or processes in the immune response not fully identified. It is important to recognize that individuals immune to one of the dengue serotypes due to a previous infection respond differently both to dengue vaccine viruses and to subsequent infection with wild-type viruses than those who are DENV naïve at time of vaccination. More importantly, persons who are dengue naïve when vaccinated may be protected partially or fully. If not, they may be at risk to enhanced infection and disease when exposed to wild-type DENV. Thus, clinical trials of DENV vaccines in dengue endemic regions are attempting to immunize two populations: dengue immunes and dengue non-immunes. Each have different efficacy and safety profiles. Since the prevalence of dengue immunity differs by age and by geography, designing clinical trials to accurately evaluate both vaccine efficacy and long-term safety poses a challenge unlike any trials of other viral vaccines. Data derived from these two immunologically different populations cannot be pooled without obscuring both efficacy and safety results [10].

Cross protection against a heterologous dengue strain occurs for several months after a dengue infection, but, later, the immunity is serotype-specific [11]. The efficacy of any dengue vaccine in the early months after infection may recapitulate this phenomenon. Months or years later, the protective efficacy and the safety profile of the two groups may diverge [12]. Waning protection by vaccine-acquired antibodies may leave those vaccinated while seronegative at risk to an enhanced disease comparable to secondary wild-type DENV infections. Appreciation of this risk resulted in inclusion in the WHO *Guidelines for Clinical Evaluation of Dengue Vaccines* of a concern “that a sub-immunogenic vaccine, or a vaccine whose efficacy wanes over time, could leave a recipient with an ‘immune profile’ which not only fails to protect, but increases the risk for experiencing severe dengue through complex immunopathological mechanisms following subsequent natural infection.” [13]. The *Guidelines* contain several recommendations designed to address this concern. The most important is that “Protection can be measured only if vaccinated and control subjects are equally at risk to mild and severe dengue.” [13]. Differences in the immunologic response to vaccination DENV-immune and non-immune individuals may result in a different efficacy and safety profile between the two groups. Future trials must take into account the effects of ADE on individuals receiving DENV vaccines and include a design that separately evaluates efficacy and safety in two immunologically different groups.

There is also an opportunity to address the issue of increased risk to individuals non-immune at vaccination in phase 4 studies and in post-marketing surveillance in countries where the Sanofi dengue vaccine has been licensed, as recommended by Hernandes-Avila et al. [14]. Additional data on the level of increased risk to vaccinees may also be obtained by longer-term surveillance of the younger populations in the phase 3 trials of the Sanofi vaccine. Post-marketing and post-trial surveillance and phase 4 studies should be designed to separately assess the effectiveness and the safety in the two immunologically different populations.
